# Stellate Ganglion Block Combined with Hyperbaric Oxygen Therapy for Sudden Sensorineural Hearing Loss: A Retrospective Three-Arm Study

**DOI:** 10.3390/life16081349

**Published:** 2026-08-17

**Authors:** Geng-He Chang, Chung-Hang Wong, An-Ting Lee, Yu-Ching Cheng, Wen-Chun Liu

**Affiliations:** 1 Department of Otolaryngology—Head and Neck Surgery, Chang Gung Memorial Hospital, Chiayi 61363, Taiwan; 2 Faculty of Medicine, College of Medicine, Chang Gung University, Taoyuan 33302, Taiwan; 3 Graduate Institute of Clinical Medical Sciences, College of Medicine, Chang Gung University, Taoyuan 33302, Taiwan; 4 Department of Anesthesiology, Chang Gung Memorial Hospital, Chiayi 613016, Taiwan; 5 Department of Anesthesiology, Star Clinic, Tainan 700017, Taiwan; 6 Department of Management, Chang Gung Memorial Hospital, Chiayi 613016, Taiwan

**Keywords:** sudden sensorineural hearing loss, stellate ganglion block, hyperbaric oxygen therapy, pure-tone audiometry, Siegel criteria, adjunctive therapy

## Abstract

Sudden sensorineural hearing loss (SSNHL) carries unpredictable recovery, and adjunctive therapies beyond systemic corticosteroids are limited. Stellate ganglion block (SGB) and hyperbaric oxygen therapy (HBOT) have each been proposed as adjuncts, yet their combined effect remains poorly characterized. A retrospective three-arm cohort study (IRB No. 202401644B0) enrolled 75 SSNHL patients: Control (standard corticosteroid, n = 39), SGB (n = 21), and SGB+HBOT (n = 15). PTA4 improvement and Siegel recovery categories were assessed at two weeks, one month, and two months. Subgroup analyses stratified patients by baseline PTA4 < 70 dB and ≥70 dB. Groups differed significantly in overall baseline PTA4 (Kruskal–Wallis *p* = 0.044; SGB group worst at 80.4 ± 17.3 dB) and in diabetes mellitus (DM) prevalence (Control 44% vs. 7% in SGB+HBOT; *p* = 0.018). Within the ≥70 dB subgroup, baseline PTA4 was balanced across groups (Control 86.5 ± 13.0 dB, SGB 88.2 ± 14.2 dB, SGB+HBOT 90.9 ± 15.3 dB; *p* = 0.748). In this balanced stratum, SGB+HBOT achieved the greatest two-month PTA4 improvement (+23.6 ± 27.2 dB) and the highest complete recovery rate (43%, 3/7), versus 27% (3/11) in Control and 9% (1/11) in SGB. No statistically significant between-group differences were identified. However, SGB+HBOT demonstrated the numerically greatest audiometric improvement and the highest complete recovery rate in the severe–profound subgroup despite baseline disadvantages, suggesting a possible therapeutic benefit for this population. These exploratory findings support prospective evaluation in an adequately powered randomized controlled trial.

## 1. Introduction

Sudden sensorineural hearing loss (SSNHL) is defined as a sensorineural hearing loss of ≥30 dB across three contiguous audiometric frequencies occurring within 72 h [1]. With an estimated annual incidence of 5–160 per 100,000 persons, SSNHL represents one of the most urgent otolaryngologic emergencies, owing to its frequently irreversible nature and its impact on patients’ quality of life [2]. Spontaneous recovery occurs in approximately 32–65% of patients, but outcomes remain highly variable and correlate with initial hearing threshold severity, audiometric configuration, patient age, and the presence of vascular comorbidities such as diabetes mellitus (DM) and hypertension [2,3]. Profound initial hearing loss (≥70 dB) portends a particularly poor prognosis, with significantly lower rates of meaningful audiological recovery [4].

High-dose systemic corticosteroids remain the cornerstone of treatment, with intratympanic corticosteroid (ITS) delivery increasingly used as primary or salvage therapy [1,5]. Despite this, a substantial proportion of patients fail to recover useful hearing, driving the search for effective adjunctive strategies. Hyperbaric oxygen therapy (HBOT) enhances cochlear oxygenation by elevating arterial partial pressure of oxygen, counteracting the ischemia and reactive oxygen species thought to underlie SSNHL pathophysiology [6]. A systematic review and meta-analysis by Joshua et al. demonstrated that HBOT combined with steroids yielded a mean hearing gain of 10.3 dB and an odds ratio of 4.3 for audiological response compared with controls [7]. More recently, Chen et al. reported that HBOT produced a significantly greater PTA4 improvement (22.7 vs. 16.2 dB, *p* = 0.007), with HBOT-treated patients 2.5 times more likely to achieve a clinically meaningful response [8]. Newth et al. reported a relative risk of 1.6 for hearing recovery with HBOT plus steroids versus steroids alone [9], and Chang et al. demonstrated that early initiation of HBOT significantly improves prognosis [10]. The 2019 AAO-HNS Clinical Practice Guideline for Sudden Hearing Loss designates HBOT as a clinical option for patients with incomplete recovery when initiated within three months of onset [11].

The stellate ganglion block (SGB), a sympathetic nerve block delivered at the cervicothoracic junction, has been proposed as an adjunct for SSNHL based on the hypothesis that cervical sympathetic overactivation produces vasospasm of the labyrinthine artery, restricting cochlear blood flow [12]. Evidence supporting SGB remains limited. Han et al., in a large retrospective study of 318 patients, found no overall benefit for SGB but noted a possible trend toward benefit in the profound hearing loss subgroup [12]. Park et al. reported a combination regimen of steroids, antivirals, anticoagulants, and SGB that achieved significantly higher hearing improvement rates (60.9% vs. 38.5%), with more pronounced effects in patients with initial hearing loss > 70 dB [13]. More recently, Li et al. demonstrated that a triple combination of SGB, ITS, and HBOT for refractory SSNHL produced significantly greater hearing gain and improved low-frequency recovery compared with ITS alone (*p* < 0.05) [14]. Oh et al. additionally reported that SGB significantly reduced depression and anxiety in patients with idiopathic SSNHL, independent of auditory recovery [15].

Despite the growing literature on each modality individually, no study has directly compared SGB alone, SGB+HBOT, and standard-care control within the same cohort stratified by hearing loss severity. The present study addresses this gap by conducting a retrospective three-arm analysis at a single tertiary center, examining audiometric outcomes at two weeks, one month, and two months post-treatment, with a primary focus on whether SGB+HBOT confers additional benefit in the severe–profound subgroup most likely to benefit from augmented cochlear perfusion.

## 2. Materials and Methods

### 2.1. Study Design and Participants

**Figure 1:**
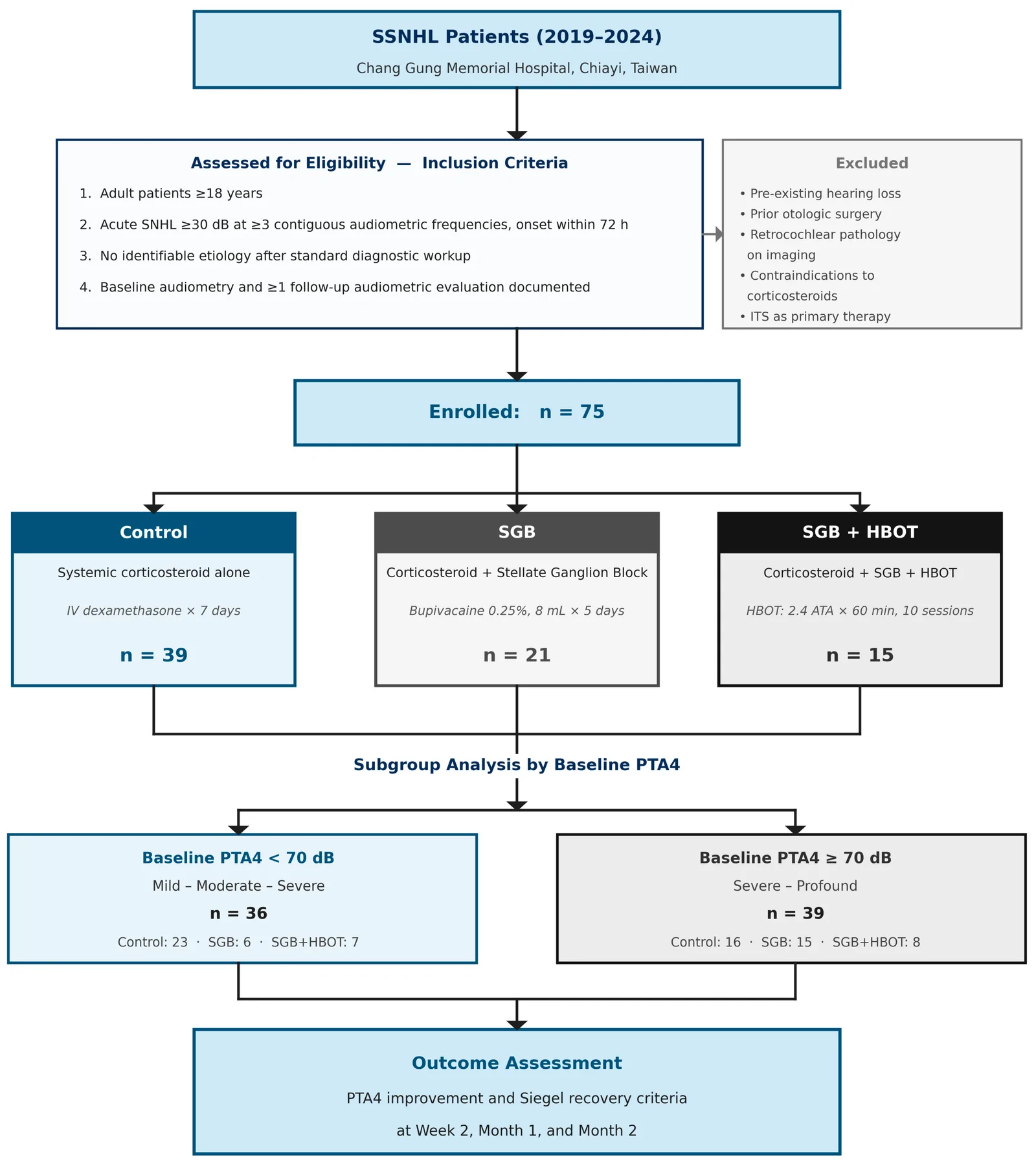
Study flowchart illustrating patient enrollment, group allocation, treatment protocols, and follow-up assessments. SSNHL, sudden sensorineural hearing loss; SGB, stellate ganglion block; HBOT, hyperbaric oxygen therapy; ITS, intratympanic steroid injection; PTA4, four-frequency pure-tone average.

This single-center retrospective cohort study was conducted at the Department of Otolaryngology–Head and Neck Surgery, Chang Gung Memorial Hospital, Chiayi, Taiwan, and was approved by the Institutional Review Board (IRB No. 202401644B0). Adult patients (≥18 years) diagnosed with SSNHL between 2019 and 2024 were eligible if they met the following criteria: (1) acute sensorineural hearing loss of ≥30 dB at three contiguous frequencies within 72 h of onset; (2) no identifiable etiology after standard diagnostic workup; and (3) documented audiometric evaluation at baseline and at least one follow-up time point. Patients with pre-existing hearing loss, prior otologic surgery, retrocochlear pathology on imaging, or contraindications to systemic corticosteroids, or who received intratympanic steroid injection as primary therapy were excluded. A total of 75 patients were enrolled: Control (standard systemic corticosteroid alone, n = 39), SGB (corticosteroid plus SGB, n = 21), and SGB+HBOT (corticosteroid plus SGB plus HBOT, n = 15) (Figure 1).

### 2.2. Treatment Protocols

All patients received systemic corticosteroids as the standard backbone therapy, administered as intravenous dexamethasone 5 mg every 8 h (Q8H) for 2 days, then 5 mg every 12 h (Q12H) for 2 days, then 5 mg once daily (QD) for 3 days (total 7-day intravenous course; cumulative dose approximately 65 mg). Patients in the SGB group underwent ultrasound-guided stellate ganglion block using 8 mL bupivacaine 0.25% at the C6 level, performed by a trained anesthesiologist under real-time ultrasound guidance following negative aspiration; successful blockade was confirmed by transient ipsilateral Horner’s syndrome. SGB was administered once daily for 5 consecutive days as a standard protocol course (5 injections per patient; some patients received fewer due to early discharge or patient preference). Patients in the SGB+HBOT group received the same SGB protocol with the addition of HBOT sessions at 2.4 atmospheres absolute (ATA) for 60 min, for 10 sessions total; the SGB injection course and HBOT sessions were conducted concurrently on the first 5 consecutive days (one injection immediately followed by one HBOT session per day), with 5 additional HBOT-only sessions completed thereafter; all treatments were initiated within two weeks of symptom onset. Treatment allocation was based on patient preference, clinical judgment, and resource availability.

Patients with pre-existing diabetes mellitus (DM) were included when systemic corticosteroid therapy was deemed clinically acceptable by the attending otolaryngologist, in consultation with the internal medicine service when indicated. As this was a retrospective study in which patients were admitted and managed by different attending physicians, no single fixed glycemic threshold was applied. The decision to redirect a patient to intratympanic steroid injection rather than systemic corticosteroids was made by the attending physician based on clinical judgment informed by pre-treatment glycemic evaluation (fasting blood glucose and HbA1c) and blood glucose measurements obtained during hospitalization. Patients who received intratympanic steroid injection as primary therapy are excluded from the present cohort, which consists exclusively of patients who received systemic (intravenous) corticosteroid therapy. All inpatients with DM receiving systemic dexamethasone had glycemic management co-supervised by the internal medicine service, with sliding-scale or basal-bolus insulin initiated as clinically indicated and blood glucose monitored at least four times daily during the inpatient course.

### 2.3. Outcome Measures

The primary outcome measure was the change in PTA4 from baseline, defined as the mean pure-tone threshold at 500, 1000, 2000, and 4000 Hz. ΔPTA4 = baseline PTA4 minus post-treatment PTA4; a positive value indicates hearing improvement. Assessments were performed at two weeks (W2), one month (M1), and two months (M2) post-treatment. Categorical outcomes were classified according to Siegel criteria, applied as mutually exclusive categories in descending order: Complete recovery (ΔPTA4 ≥ 25 dB or final PTA4 ≤ 25 dB), Marked recovery (ΔPTA4 ≥ 15 dB), Partial recovery (ΔPTA4 ≥ 10 dB), and No recovery (ΔPTA4 < 10 dB) [16]. Subgroup analyses stratified patients by baseline PTA4: mild–moderate–severe (<70 dB) and severe–profound (≥70 dB).

### 2.4. Statistical Analysis

Continuous variables were compared using the Kruskal–Wallis test with Mann–Whitney U pairwise comparisons for non-normally distributed data, and one-way ANOVA for normally distributed data. Categorical variables were compared using Chi-square or Fisher’s exact test as appropriate. All tests were two-tailed (α = 0.05). Analysis of covariance (ANCOVA) was performed to estimate group-adjusted means for PTA4 improvement at each follow-up timepoint, with covariates: baseline PTA4, age, diabetes mellitus (DM) status, and hypertension; adjusted means with 95% confidence intervals (CIs) are reported. A post hoc power analysis was conducted for the primary comparison of interest in the severe subgroup (SGB+HBOT vs. SGB at 2 months). Pairwise comparisons are reported with both uncorrected and Bonferroni-corrected *p*-values (multiplied by 3, capped at 1.0); all comparisons are considered exploratory. A sensitivity analysis using last observation carried forward (LOCF) compared complete-case results to all-available-data results to assess the impact of differential follow-up attrition (Supplementary Table S4). Effect sizes are expressed as Cohen’s d with 95% CIs. Statistical analyses were performed using Python 3 (NumPy and pandas libraries). Onset-to-treatment interval was compared across groups using the Kruskal–Wallis test; pairwise post hoc comparisons were performed using the Mann–Whitney U test with Bonferroni correction.

## 3. Results

### 3.1. Patient Demographics and Baseline Characteristics

Seventy-five patients were included: Control (n = 39), SGB (n = 21), and SGB+HBOT (n = 15). Baseline characteristics are summarized in Table 1. Mean age was similar across groups (Control 55.9 ± 16.4 years, SGB 56.3 ± 13.3 years, SGB+HBOT 56.7 ± 8.9 years; *p* = 0.99). Sex distribution did not differ significantly (Control 51% male, SGB 71%, SGB+HBOT 53%; *p* = 0.28), nor did hypertension prevalence (Control 23%, SGB 24%, SGB+HBOT 27%; *p* = 0.96).

Two baseline imbalances were identified. First, DM prevalence differed significantly: 44% in Control, 29% in SGB, and 7% in SGB+HBOT (*p* = 0.018, Fisher’s exact test). DM is a recognized negative prognostic factor for SSNHL recovery [ ,, constituting a potential confounding variable in overall comparisons (see Discussion). Second, overall baseline PTA4 differed significantly across groups (Kruskal–Wallis *p* = 0.044): Control 63.7 ± 24.4 dB, SGB 80.4 ± 17.3 dB, SGB+HBOT 66.7 ± 29.0 dB.

Within the ≥70 dB subgroup, baseline PTA4 was well balanced: Control 86.5 ± 13.0 dB, SGB 88.2 ± 14.2 dB, SGB+HBOT 90.9 ± 15.3 dB (Kruskal–Wallis *p* = 0.748; all pairwise *p* > 0.42). Notably, the SGB+HBOT group entered this stratum with the highest mean baseline threshold. Within the <70 dB subgroup, baseline PTA4 differed significantly (Control 47.8 ± 17.4 dB, SGB 60.8 ± 6.3 dB, SGB+HBOT 38.9 ± 12.3 dB; *p* = 0.013; SGB vs. SGB+HBOT *p* = 0.002), limiting direct comparisons in this stratum.

Follow-up retention was high. At two weeks, 95%, 100%, and 100% of Control, SGB, and SGB+HBOT patients completed evaluation. At two months, retention was 64% (25/39), 71% (15/21), and 93% (14/15), respectively. A LOCF sensitivity analysis confirmed directionally consistent results across groups (Supplementary Table S4).

Audiogram configuration was classifiable for all 75 patients using frequency-specific threshold data (500, 1000, 2000, 4000 Hz). Flat configuration was most common across all groups: Control 26 (67%), SGB 11 (52%), SGB+HBOT 7 (47%). Low-frequency configuration was present in 5 (13%), 1 (5%), and 3 (20%) patients; high-frequency sloping in 2 (5%), 2 (10%), and 1 (7%); and profound pattern in 6 (15%), 7 (33%), and 4 (27%), respectively. The distribution of audiogram configuration did not differ significantly across groups (χ^2^ = 5.142, df = 6, *p* = 0.526; Table 1).

Time from symptom onset to treatment initiation differed significantly across groups: Control median 5 days (range 0–21), SGB median 10 days (range 3–31), and SGB+HBOT median 14 days (range 4–90; Kruskal–Wallis H = 21.26, *p* < 0.001; Supplementary Table S6). The SGB+HBOT group had the longest onset-to-treatment interval, yet demonstrated the numerically best 2-month audiometric outcome after covariate adjustment, suggesting that treatment timing disadvantage may have attenuated rather than inflated the observed effect.

### 3.2. Audiometric Outcomes by Subgroup

**Figure 2:**
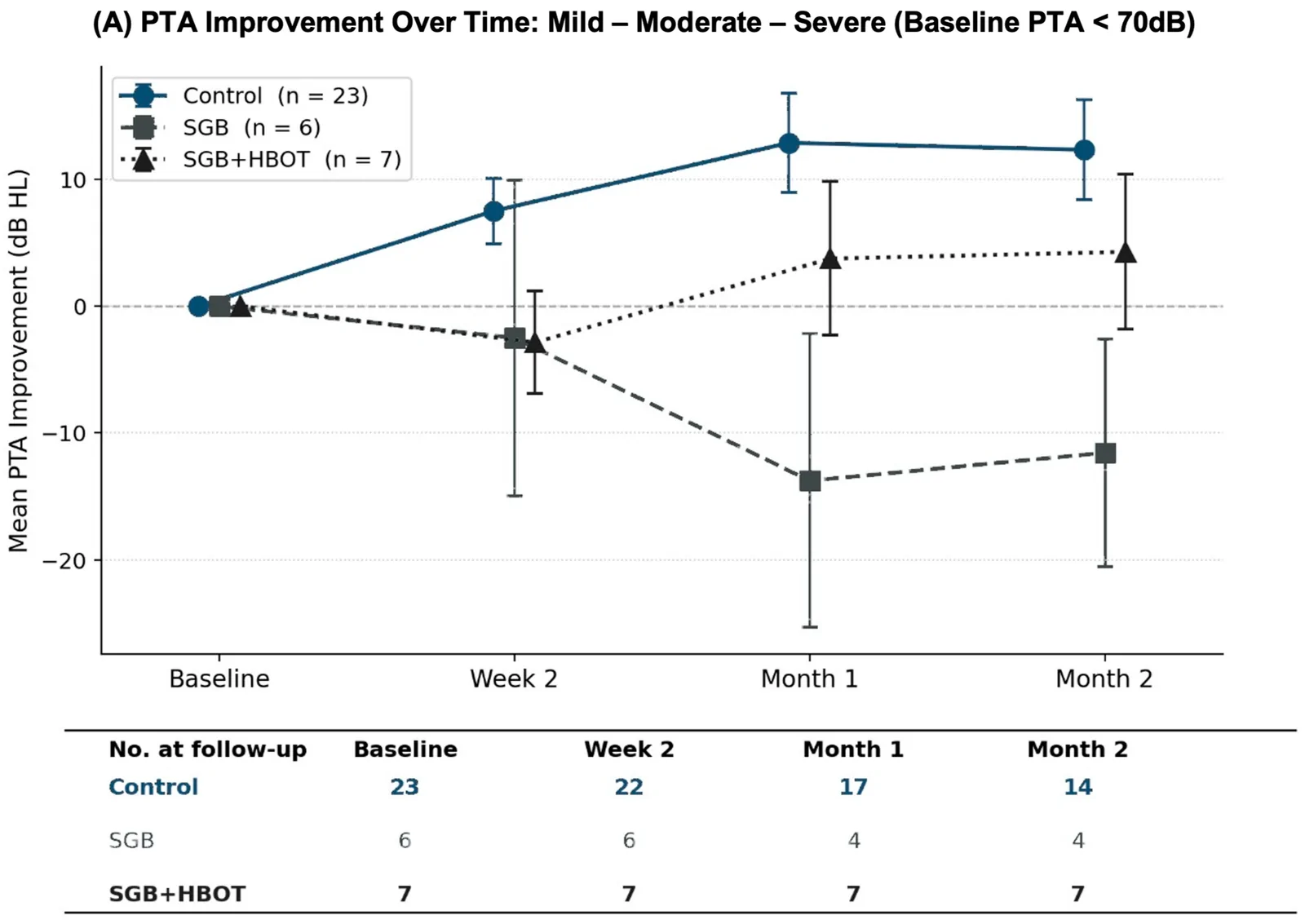

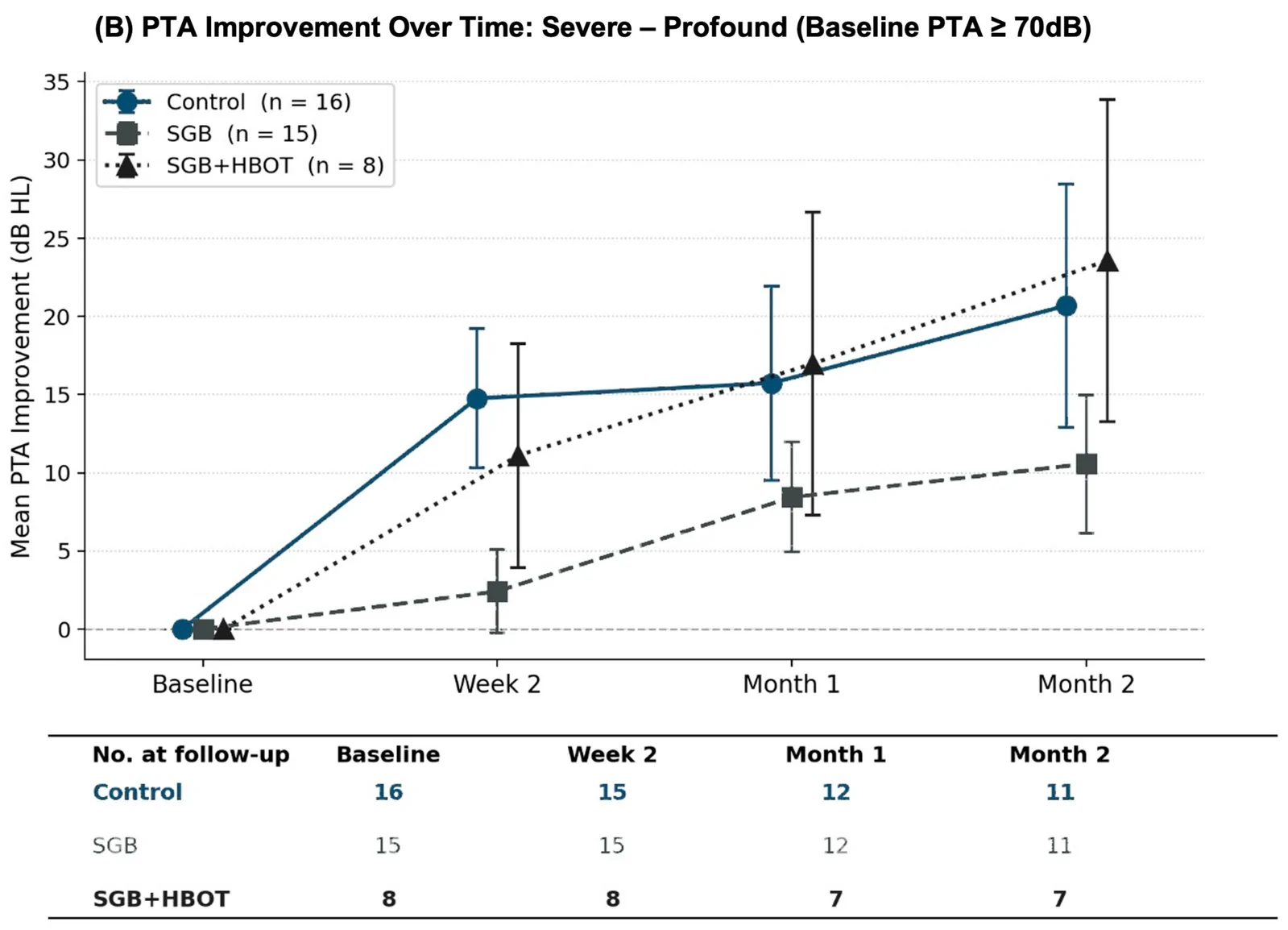
Pure-Tone Average (PTA) improvement over time stratified by initial hearing loss severity. (**A**) PTA improvement in patients with mild-to-severe initial hearing loss (Baseline PTA < 70 dB). (**B**) PTA improvement in patients with severe-to-profound initial hearing loss (Baseline PTA ≥ 70 dB). Data points represent group means ± 95% confidence intervals (95% CI) at 2 weeks, 1 month, and 2 months. PTA4, four-frequency pure-tone average; SGB, stellate ganglion block; HBOT, hyperbaric oxygen therapy; CI, confidence interval.

All primary analyses were stratified by severity subgroup (Figure 2A,B). Within the ≥70 dB subgroup, confirmed baseline PTA4 balance (*p* = 0.748) supports valid between-group comparisons.

Pairwise comparisons at the two-month endpoint showed no statistically significant differences after Bonferroni correction in either subgroup (Supplementary Table S3). In the mild–moderate subgroup (<70 dB), Control vs. SGB showed a nominally significant difference (uncorrected *p* = 0.038; Bonferroni *p* = 0.114; n = 14 vs. n = 4) that did not survive correction; this comparison involved only four SGB patients and should be interpreted cautiously. In the severe–profound subgroup (≥70 dB), all pairwise comparisons were non-significant with or without Bonferroni correction (all uncorrected *p* > 0.25).

Effect sizes were small to medium with wide confidence intervals crossing zero: Cohen’s d for Control vs. SGB across all patients was 0.581 (95% CI −0.072 to 1.234); in the severe subgroup, SGB vs. SGB+HBOT d = −0.640 (95% CI −1.693 to 0.412; negative sign indicates greater SGB+HBOT improvement) and Control vs. SGB d = 0.482 (95% CI −0.421 to 1.386) (Supplementary Table S5).

### 3.3. Recovery Outcomes by Siegel Criteria

**Figure 3:**
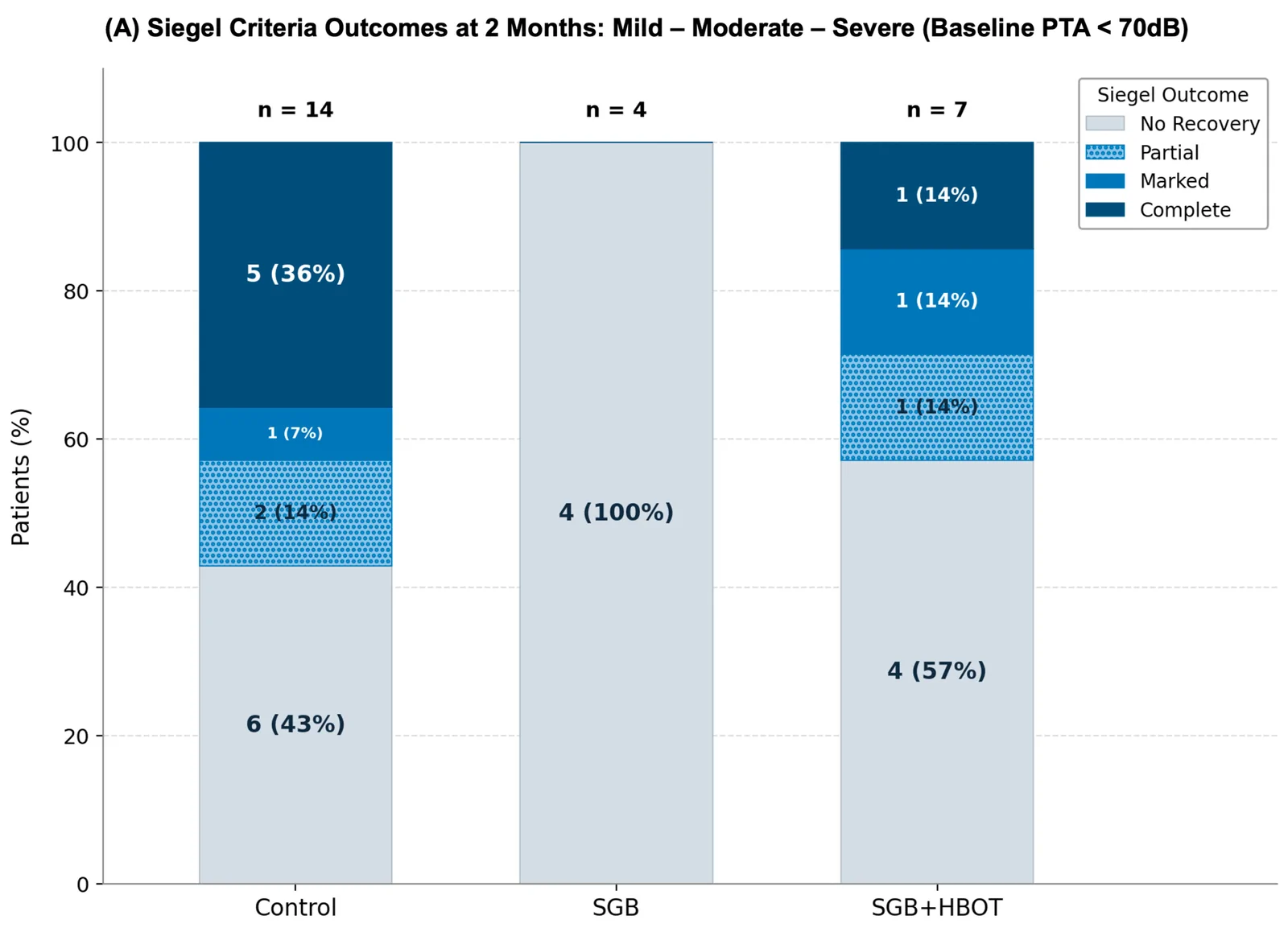

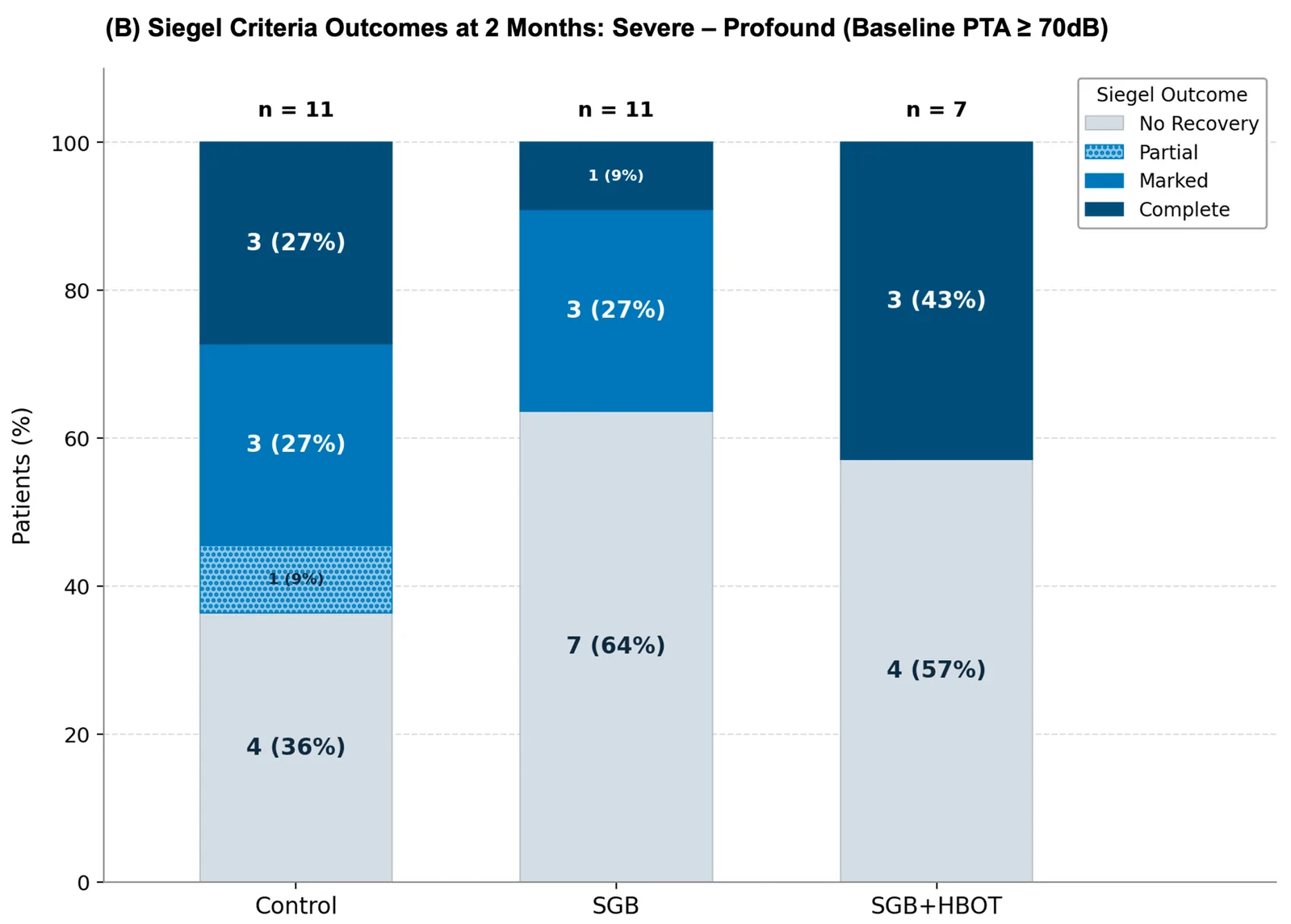
(**A**) Siegel recovery categories at two months in the mild-to-moderate–severe subgroup (PTA4 < 70 dB; Control n = 14, SGB n = 4, SGB+HBOT n = 7). Categories are mutually exclusive in descending order: Complete (ΔPTA4 ≥ 25 dB or final ≤25 dB), Marked (ΔPTA4 ≥ 15 dB), Partial (ΔPTA4 ≥ 10 dB), No Recovery (ΔPTA4 < 10 dB). Baseline PTA4 was not balanced (*p* = 0.013). No significant differences (Fisher’s exact test: all *p* ≥ 0.245). PTA4, four-frequency pure-tone average; SGB, stellate ganglion block; HBOT, hyperbaric oxygen therapy. (**B**) Siegel recovery categories at two months in the severe–profound subgroup (PTA4 ≥ 70 dB; Control n = 11, SGB n = 11, SGB+HBOT n = 7). Baseline PTA4 was balanced (*p* = 0.748). SGB+HBOT achieved the highest complete recovery rate (43%, 3/7) versus Control (27%) and SGB (9%), despite the highest mean baseline (90.9 dB). The SGB+HBOT distribution shows a bimodal pattern (43% complete, 57% no recovery, 0% intermediate). No significant differences (Fisher’s exact test: all *p* ≥ 0.670).

Recovery distributions at two months are illustrated in Figure 3A,B.

In the < 70 dB subgroup (at two months: Control n = 14, SGB n = 4, SGB+HBOT n = 7), complete recovery was achieved in 36% (5/14) of Control, 0% (0/4) of SGB, and 14% (1/7) of SGB+HBOT. No recovery was documented in 43% (6/14), 100% (4/4), and 57% (4/7). No significant differences were found (Fisher’s exact test: all *p* ≥ 0.245).

In the ≥70 dB subgroup (at two months: Control n = 11, SGB n = 11, SGB+HBOT n = 7), SGB+HBOT achieved the highest complete recovery rate at 43% (3/7), compared with 27% (3/11) in Control and 9% (1/11) in SGB. A striking feature of the SGB+HBOT distribution is its polarized pattern: 43% complete recovery and 57% (4/7) no recovery, with 0% in the intermediate marked or partial categories. This contrasts with Control (complete 27%, marked 27%, partial 9%, no recovery 36%) and SGB (complete 9%, marked 27%, no recovery 64%), both of which show more graded distributions. This pattern accounts for why the group means are numerically close (+23.6 vs. +20.7 dB): the SGB+HBOT distribution is bimodal, driven by strong responders and non-responders. No statistically significant between-group differences were identified (Fisher’s exact test: all *p* ≥ 0.670).

## 4. Discussion

This retrospective three-arm study evaluated SGB and HBOT as adjuncts to standard corticosteroid treatment for SSNHL. The principal finding is that SGB+HBOT demonstrated the highest complete recovery rate (43%, 3/7) in the severe–profound subgroup (≥70 dB), within a stratum where baseline PTA4 was confirmed to be balanced and where SGB+HBOT entered at a numerical audiometric disadvantage. The polarized, bimodal recovery pattern in SGB+HBOT—with 43% complete recovery and 57% no recovery, and no intermediate outcomes—contrasts with the more graded distributions in Control and SGB, suggesting that the treatment combination may produce a qualitatively distinct therapeutic response rather than a uniform mean shift.

Within the ≥70 dB subgroup, baseline PTA4 was well balanced across all three groups (*p* = 0.748), and the SGB+HBOT group had the highest mean baseline threshold (90.9 dB), confirming that the outcome advantage cannot be attributed to a more favorable audiometric starting point. This is a key methodological strength supporting the validity of between-group comparisons in this stratum.

The rationale for combining SGB and HBOT stems from complementary mechanisms. SGB interrupts cervical sympathetic tone, theoretically relieving vasospasm of the labyrinthine artery and improving cochlear microcirculation [12]. HBOT elevates perilymphatic oxygen tension, counteracting cochlear ischemia and attenuating oxidative stress [6]. Together, they may address both the vascular and metabolic components of cochlear injury synergistically, consistent with Li et al.’s demonstration that SGB+ITS+HBOT triple therapy produced significantly greater hearing gain and low-frequency recovery versus ITS alone (*p* < 0.05) [14].

HBOT’s efficacy as a SSNHL adjunct is supported by a growing evidence base. Joshua et al. reported a mean hearing gain of 10.3 dB and an odds ratio of 4.3 for audiological response with HBOT [7]. Chen et al. demonstrated significantly greater PTA4 improvement with HBOT (22.7 vs. 16.2 dB, *p* = 0.007) and a 2.5-fold higher likelihood of clinically meaningful response [8]. Newth et al. reported a relative benefit of 1.6 for hearing recovery with HBOT plus steroids versus steroids alone [9]. Meliante et al.’s systematic review identified ITS+HBOT as among the most effective salvage regimens [5]. Wang et al. demonstrated that early HBOT initiation significantly improves prognosis [10]. The 2019 AAO-HNS Clinical Practice Guideline for SSNHL designates HBOT as an option for patients with incomplete recovery within three months of onset [11]. Choi et al. retrospectively analyzed 2206 patients (113 in matched analysis), finding no significant overall improvement with HBOT but a significant benefit in the DM subgroup [17]. This selective benefit in DM patients is relevant: the high DM prevalence in the Control group (44%) may have attenuated recovery there, while the absence of DM in the SGB+HBOT group (7%) may have limited HBOT’s DM-related benefit in that arm.

SGB alone yielded the lowest mean two-month improvement (+10.6 dB) (Supplementary Figure S1) and the lowest complete recovery rate (9%, 1/11) in the ≥70 dB subgroup, consistent with Han et al.’s finding of no overall SGB benefit in 318 patients [12]. The SGB group also presented with higher baseline hearing loss (median PTA_4_ 75.0 dB) compared with the Control group (median PTA_4_ 62.5 dB), and a longer onset-to-treatment interval (median 10 vs. 5 days), suggesting a more severely affected population with inherently lower spontaneous recovery potential. Park et al.’s combination regimen including SGB achieved higher overall improvement rates (60.9% vs. 38.5%), with more pronounced effects in patients with hearing loss > 70 dB [13]. Together, these data suggest SGB alone may be insufficient as a cochlear adjunct, but may provide synergistic benefit when combined with HBOT.

The DM imbalance (Control 44%, SGB+HBOT 7%; *p* = 0.018) requires careful interpretation. To quantify the impact of DM confounding, ANCOVA was performed adjusting for baseline PTA4, age, DM, and hypertension. After adjustment, SGB+HBOT retained the numerically greatest 2-month improvement (adjusted mean 18.64 dB, 95% CI 8.42–28.87) compared with Control (14.87 dB, 95% CI 7.22–22.53) and SGB (2.14 dB, 95% CI −7.63 to 11.91), suggesting the DM imbalance does not fully account for the observed pattern (Supplementary Table S1). Nevertheless, residual DM-related confounding cannot be excluded. Prospective randomized studies with DM-stratified allocation are needed to isolate the true treatment effect.

Onset-to-treatment interval also differed significantly across groups (Control median 5 days vs. SGB+HBOT median 14 days; Kruskal–Wallis *p* < 0.001; Supplementary Table S6). Shorter time to treatment is an established positive prognostic factor for SSNHL, meaning the SGB+HBOT group faced an additional baseline disadvantage beyond DM imbalance. The fact that this group nonetheless achieved the numerically greatest audiometric improvement and the highest complete recovery rate in the severe–profound subgroup suggests that the observed benefit, if real, was likely attenuated rather than inflated by differential treatment timing.

Oh et al. reported significant reductions in BDI and GAD-7 scores following SGB in SSNHL patients, independent of hearing recovery [15], suggesting neuropsychological benefits of SGB-containing regimens that audiometric endpoints alone cannot capture.

Several limitations merit emphasis. The retrospective non-randomized design precludes causal inference. A post hoc power analysis for the primary comparison (SGB+HBOT vs. SGB in the severe subgroup at 2 months; observed Cohen’s d = 0.640, n = 7 vs. 11) estimated observed power at approximately 21% (α = 0.05, two-tailed; Supplementary Table S2), confirming the cohort was substantially underpowered; an adequately powered RCT would require approximately 38 participants per arm. Subgroup sizes were small at 2 months (7–14 per group), and the 43% complete recovery estimate (3/7) in SGB+HBOT would fall to 29% with one fewer responder. Differential follow-up attrition was observed (Control 64%, SGB 71%, SGB+HBOT 93% at 2 months); a sensitivity analysis confirmed directionally consistent results across both complete-case and all-available-data approaches (Supplementary Table S4). Effect sizes were small to medium across primary comparisons (Cohen’s d range 0.110–0.640), with wide 95% CIs crossing zero, accurately reflecting the statistical uncertainty inherent in this small cohort; all pairwise comparisons are considered exploratory (Supplementary Table S5). Although ANCOVA adjusting for baseline PTA4, age, DM, and hypertension yielded directionally consistent results, residual confounding cannot be excluded in this non-randomized design. A linear mixed-effects model (LMM) with time-by-treatment interaction would more appropriately handle within-patient correlation across repeated audiometric measurements; however, LMM was not applied in the present study owing to the limited sample size, which was insufficient to reliably estimate the between-subject random effects required by this approach. Future prospective studies should incorporate LMM as the primary analytic framework. These limitations are being addressed in an ongoing prospective randomized controlled trial.

## 5. Conclusions

This retrospective three-arm cohort study found that SGB+HBOT was associated with the numerically greatest audiometric improvement and the highest complete recovery rate in the severe–profound SSNHL subgroup, despite notable baseline disadvantages in DM prevalence and onset-to-treatment interval. No statistically significant between-group differences were identified, and residual confounding from this non-randomized design cannot be excluded. Nonetheless, the observed pattern suggests that SGB+HBOT may offer a therapeutic benefit for severe SSNHL. These hypothesis-generating findings warrant prospective evaluation in an adequately powered randomized controlled trial.

## Figures and Tables

**Table 1:** Baseline characteristics of patients with sudden sensorineural hearing loss.

Variable	Control (n = 39)	SGB (n = 21)	SGB+HBOT (n = 15)	*p*-Value
**Age (years)**				
Mean ± SD	55.9 ± 16.4	56.3 ± 13.3	56.7 ± 8.9	0.99 †
Range	19–80	31–80	40–72	
**Sex**				
Male	20 (51%)	15 (71%)	8 (53%)	0.28 ‡
**Comorbidities**				
Hypertension	9 (23%)	5 (24%)	4 (27%)	0.96 ‡
Diabetes mellitus	17 (44%)	6 (29%)	1 (7%)	0.018 * ‡
**Pre-treatment PTA4 (dB HL)**				
Mean ± SD	63.7 ± 24.4	80.4 ± 17.3	66.7 ± 29.0	0.044 * †
Median (IQR)	65.0 (46–83)	80.0 (68–90)	68.8 (42–92)	
**Hearing loss severity**				
Mild (≤40 dB)	5 (13%)	0 (0%)	3 (20%)	
Moderate (41–60 dB)	12 (31%)	2 (10%)	4 (27%)	
Severe (61–90 dB)	17 (44%)	14 (67%)	4 (27%)	
Profound (>90 dB)	5 (13%)	5 (24%)	4 (27%)	
**Audiogram configuration**				
Flat	26 (67%)	11 (52%)	7 (47%)	0.664 ‡
Low-frequency	5 (13%)	1 (5%)	3 (20%)	
High-frequency sloping	2 (5%)	1 (5%)	1 (7%)	
Profound pattern	2 (5%)	3 (14%)	1 (7%)	
**Follow-up completion**				
Week 2	37 (95%)	21 (100%)	15 (100%)	
≥1 month	29 (74%)	16 (76%)	14 (93%)	
≥2 months	25 (64%)	15 (71%)	14 (93%)	

PTA4, four-frequency pure-tone average (500, 1000, 2000, 4000 Hz); SGB, stellate ganglion block; HBOT, hyperbaric oxygen therapy; IQR, interquartile range. † Kruskal–Wallis test. ‡ Chi-square or Fisher’s exact test. * *p* < 0.05. Audiogram configuration classified from frequency-specific threshold data (500, 1000, 2000, 4000 Hz) available for 63 of 75 patients (Control n = 35, SGB n = 16, SGB+HBOT n = 12); percentages are based on group total n.

## Data Availability

The data presented in this study are available on request from the corresponding author.

## Supplementary Materials

The following supporting information can be downloaded at: https://www.mdpi.com/article/10.3390/life16081349/s1, Figure S1. PTA4 Improvement from Baseline Severe-Profound (Baseline PTA4 > 70 dB); Table S1. ANCOVA-Adjusted Mean PTA4 Improvement by Group and Timepoint; Table S2. Post-Hoc Statistical Power Analysis; Table S3. Bonferroni-Corrected Pairwise Comparisons at Primary 2-Month Endpoint Table S4. LOCF Sensitivity Analysis: PTA4 Improvement at 2 Months; Table S5. Cohen’s d Effect Sizes for PTA4 Improvement at 2 Months; Table S6. Onset-to-Treatment Intervals by Group.
