# Supplementary material for: Stellate Ganglion Block Combined with Hyperbaric Oxygen Therapy for Sudden Sensorineural Hearing Loss: A Retrospective Three-Arm Study
Source: Life (Basel). 2026 Aug 17;16(8):1349. doi: 10.3390/life16081349 (PMC13514495; doi:10.3390/life16081349)
Supplement: Supplementary file 1 [file life-16-01349-s001.zip › Supplementary_Table_S1_ANCOVA.pdf]

Supplementary Table S1. ANCOVA-Adjusted Mean PTA4 Improvement by Group and Timepoint

Timepoint	Control Adj. Mean (95% CI)	SGB Adj. Mean (95% CI)	SGB+HBOT Adj. Mean (95% CI)	*F*	*p*	η^2p
2 Weeks	10.57 dB (5.56–15.58)	−0.66 dB (−7.32–6.01)	6.59 dB (−1.35–14.54)	4.92	<0.001	0.14
1 Month	13.44 dB (6.76–20.12)	1.32 dB (−7.66–10.30)	13.42 dB (3.82–23.02)	3.82	<0.001	0.11
2 Months	14.87 dB (7.22–22.53)	2.14 dB (−7.63–11.91)	**18.64 dB (8.42–28.87)**	3.57	<0.001	0.12

Abbreviations: PTA4 = four-frequency pure-tone average (500, 1000, 2000, 4000 Hz); CI = confidence interval; η^2p = partial eta-squared effect size.

ANCOVA model: outcome = PTA4 improvement from baseline (positive = improvement); covariates = baseline PTA4, age, diabetes mellitus status (yes/no), hypertension status (yes/no). Sex was evaluated as a covariate but did not meaningfully alter group estimates and was excluded from the final model to conserve degrees of freedom.

Adjusted means represent predicted group values at covariate means. Bold values indicate the numerically largest improvement at 2 months.

p-values represent the overall group effect (F-test) from the ANCOVA. All comparisons are exploratory; no pairwise significance is implied.

Sample sizes at 2 months: Control n=25, SGB n=15, SGB+HBOT n=14.
