# Supplementary material for: Stellate Ganglion Block Combined with Hyperbaric Oxygen Therapy for Sudden Sensorineural Hearing Loss: A Retrospective Three-Arm Study
Source: Life (Basel). 2026 Aug 17;16(8):1349. doi: 10.3390/life16081349 (PMC13514495; doi:10.3390/life16081349)
Supplement: Supplementary file 1 [file life-16-01349-s001.zip › Supplementary_Table_S2_Power.pdf]

Supplementary Table S2. Post-Hoc Statistical Power Analysis

Parameter	Value
**Comparison of interest**	SGB+HBOT vs. SGB (severe subgroup, PTA4 ≥70 dB, 2-month improvement)
**Observed Cohen's d**	0.640 (medium–large effect)
**Sample size**	n_1 = 7 (SGB+HBOT), n_2 = 11 (SGB)
**α (two-tailed)**	0.05
**Observed power (1 − β)**	≈ 21%
**Required n per arm for 80% power**	≈ 38 participants per arm (severe-subgroup stratum)
**Required n per arm for 90% power**	≈ 52 participants per arm
**Interpretation**	The study was severely underpowered for detecting the observed effect. A definitive RCT would require substantially larger sample sizes.

Power analysis performed using two-sample t-test formula for independent groups: power = 1 − β, where β is determined by the non-central t-distribution.

The most clinically important comparison — SGB+HBOT vs. SGB in the severe subgroup — was selected as the primary comparison for power estimation given the therapeutic hypothesis that combined therapy is most beneficial in severe cases.

Cohen's d = 0.640 was computed from the observed 2-month PTA4 improvement means and pooled standard deviation in the complete-case sample (SGB+HBOT: n=7, mean=23.57 dB; SGB: n=11, mean=10.57 dB).

Sample size estimates are for the severe-subgroup stratum only and assume equal allocation, α = 0.05 (two-tailed), and a similar effect size in the target population.
