# Supplementary material for: Stellate Ganglion Block Combined with Hyperbaric Oxygen Therapy for Sudden Sensorineural Hearing Loss: A Retrospective Three-Arm Study
Source: Life (Basel). 2026 Aug 17;16(8):1349. doi: 10.3390/life16081349 (PMC13514495; doi:10.3390/life16081349)
Supplement: Supplementary file 1 [file life-16-01349-s001.zip › Supplementary_Table_S3_Bonferroni.pdf]

Supplementary Table S3. Bonferroni-Corrected Pairwise Comparisons at Primary 2-Month Endpoint

Subgroup	Comparison	Timepoint	Uncorrected p	Bonferroni p	Significant?
<70 dB	Control vs. SGB	2 months	0.038	0.114	No
	Control vs. SGB+HBOT	2 months	0.786	1.000	No
	SGB+HBOT vs. SGB	2 months	0.250	0.751	No
≥70 dB	Control vs. SGB	2 months	0.275	0.826	No
	Control vs. SGB+HBOT	2 months	0.827	1.000	No
	SGB+HBOT vs. SGB	2 months	0.279	0.838	No

Abbreviations: dB = decibels hearing level; p = p-value.

Bonferroni correction: corrected p = uncorrected p × 3 (number of pairwise comparisons per subgroup), capped at 1.0.

Uncorrected p-values derived from Mann-Whitney U tests for pairwise comparisons at the 2-month PTA4 improvement endpoint.

After correction, no pairwise comparison achieves statistical significance in either subgroup. The one nominally significant comparison (Control vs. SGB in the <70 dB subgroup, uncorrected p = 0.038) becomes non-significant after correction (p = 0.114).

All analyses are exploratory; Bonferroni correction may be conservative given correlation among comparisons.
