# Supplementary material for: Stellate Ganglion Block Combined with Hyperbaric Oxygen Therapy for Sudden Sensorineural Hearing Loss: A Retrospective Three-Arm Study
Source: Life (Basel). 2026 Aug 17;16(8):1349. doi: 10.3390/life16081349 (PMC13514495; doi:10.3390/life16081349)
Supplement: Supplementary file 1 [file life-16-01349-s001.zip › Supplementary_Table_S4_LOCF.pdf]

Supplementary Table S4. LOCF Sensitivity Analysis: PTA4 Improvement at 2 Months

Group	CC (n)	CC Mean ± SD (dB)	LOCF (n)	LOCF Mean ± SD (dB)	Δ Mean	Direction	Consistent?
**All patients**							
Control	25	16.00 ± 20.34	37	15.88 ± 18.87	−0.12	Same	Yes
SGB	15	4.67 ± 18.01	21	5.54 ± 17.38	+0.87	Same	Yes
SGB+HBOT	14	13.93 ± 23.72	15	13.25 ± 23.01	−0.68	Same	Yes
**Severe subgroup (PTA4 ≥70 dB)**							
Control	11	20.68	15	21.83	+1.15	Same	Yes
SGB	11	10.57	15	7.50	−3.07	Same	Yes
SGB+HBOT	7	23.57	8	21.09	−2.48	Same	Yes

Abbreviations: CC = complete-case analysis (patients with 2-month audiometric data only); LOCF = last observation carried forward; SD = standard deviation; Δ Mean = LOCF mean minus CC mean.

LOCF method: for patients missing 2-month data, the last available follow-up PTA4 was substituted (1-month if available, otherwise 2-week). Improvement = baseline PTA4 minus follow-up PTA4 (positive values = improvement).

"Same" direction indicates that the group ranking (Control > SGB+HBOT > SGB in improvement) is preserved between CC and LOCF analyses.

SD not shown for severe subgroup due to small n (7–15 per group). The close agreement between CC and LOCF estimates suggests that differential loss to follow-up did not substantially alter the observed pattern of results.
