# Supplementary material for: Stellate Ganglion Block Combined with Hyperbaric Oxygen Therapy for Sudden Sensorineural Hearing Loss: A Retrospective Three-Arm Study
Source: Life (Basel). 2026 Aug 17;16(8):1349. doi: 10.3390/life16081349 (PMC13514495; doi:10.3390/life16081349)
Supplement: Supplementary file 1 [file life-16-01349-s001.zip › Supplementary_Table_S5_CohenD.pdf]

Supplementary Table S5. Cohen's d Effect Sizes for PTA4 Improvement at 2 Months

Comparison	n_1 vs n_2	Cohen's d	95% CI	Interpretation
**All patients (2-month PTA4 improvement, complete-case)**				
Control vs. SGB	25 vs 15	0.581	−0.072 to 1.234	Medium–large (d ≥ 0.5); Control better; NS after Bonferroni
Control vs. SGB+HBOT	25 vs 14	0.096	−0.559 to 0.751	Small (d < 0.2); negligible difference; NS
SGB vs. SGB+HBOT	15 vs 14	−0.442	−1.214 to 0.330	Medium (|d| ≥ 0.4); SGB+HBOT numerically better; NS
**Severe subgroup (baseline PTA4 ≥70 dB, 2-month PTA4 improvement, complete-case)**				
Control vs. SGB	11 vs 11	0.482	−0.421 to 1.386	Medium (d ≥ 0.4); Control better; NS
Control vs. SGB+HBOT	11 vs 7	−0.110	−1.135 to 0.916	Small; negligible difference; NS
SGB vs. SGB+HBOT	11 vs 7	−0.640	−1.693 to 0.412	Medium–large (|d| ≥ 0.5); SGB+HBOT higher; NS

Abbreviations: PTA4 = four-frequency pure-tone average; CI = confidence interval; NS = not statistically significant after Bonferroni correction; dB = decibels.

Cohen's d computed using pooled standard deviation formula: $d = (M_1 - M_2) / SD\_pooled$. Positive d indicates greater improvement in Group 1; negative d indicates greater improvement in Group 2.

95% CIs computed using non-central t-distribution approximation. Wide CIs reflect small sample sizes and should be interpreted cautiously.

Conventional Cohen's d thresholds: small ≥ 0.2, medium ≥ 0.5, large ≥ 0.8. All comparisons are exploratory.

Hodges-Lehmann estimates were not computed in this analysis; Cohen's d with 95% CI is reported as the primary effect size metric.
