# Supplementary material for: Stellate Ganglion Block Combined with Hyperbaric Oxygen Therapy for Sudden Sensorineural Hearing Loss: A Retrospective Three-Arm Study
Source: Life (Basel). 2026 Aug 17;16(8):1349. doi: 10.3390/life16081349 (PMC13514495; doi:10.3390/life16081349)
Supplement: Supplementary file 1 [file life-16-01349-s001.zip › Supplementary_Table_S6_Onset.pdf]

Supplementary Table S6. Onset-to-Treatment Intervals by Group

Group	n	Median (days)	Mean ± SD (days)	Range (days)	Comment
Control	39	5	5.9 ± 4.6	0–21	Earliest treatment; shortest interval
SGB	18	10	10.9 ± 6.3	3–31	Intermediate interval
SGB+HBOT	14	14	21.6 ± 22.6	4–90	Longest interval; yet numerically best 2-month outcome
**Kruskal-Wallis**	—	—	**H = 21.26**	**p < 0.001**	Significant between-group difference in onset-to-treatment

Abbreviations: SD = standard deviation; n = number of patients with available onset date; H = Kruskal-Wallis test statistic.

Onset-to-treatment interval = number of days from symptom onset date to first treatment date. For the Control group, treatment date = hospital admission date for intravenous corticosteroid initiation. For the SGB and SGB+HBOT groups, treatment date = date of first SGB procedure (which also coincided with first HBOT session for the SGB+HBOT group).

SGB group n=18 (vs. baseline n=21): 3 patients had missing onset date in the raw dataset and were excluded from this analysis.

SGB+HBOT group n=14 (vs. baseline n=15): 1 patient had missing onset date.

The SGB+HBOT group had the longest onset-to-treatment interval (median 14 days, range 4–90 days) despite demonstrating the numerically best 2-month audiometric improvement after covariate adjustment. Since earlier treatment is a known positive prognostic factor in SSNHL, this pattern suggests the treatment timing disadvantage in SGB+HBOT may have attenuated rather than inflated the observed improvement.
